# The Minneapolis Board of Trade Milk and Meat Inspection Meeting

**Published:** 1895-06

**Authors:** 


### EDUCATIONAL.

The Board of Trade of Minneapolis held a meeting on the 1st of April to direct public attention to the defects of the present system of milk and dairy inspection and the presence of bovine tuberculosis.

Dr. Charles E. Cotton, V.M.D., addressed the Board on the value of tuberculin as a diagnostic agent, and the absence of danger from its use when properly employed. He reported three herds tested by him at the solicitation of the State Board of Health. In a total of seventy-one cattle twenty-seven reacted.

Dr. M. H. Reynolds, Professor of Veterinary Science in the State University, addressed the meeting on the importance of some action being taken. Speaking of infected animals, he said that if infected at all they may be infectious and may be a dangerous factor in the stable. He reported that experiments with tuberculin were being made at the State experiment station, not only as a diagnostic agent, but also to determine better whether it has not curative properties in conjunction with other helpful environments. He further expressed the opinion, from close study and careful observation, that the presence of tuberculosis was not so largely a condition of breed as of individuality and surrounding conditions. He advised the testing of all herds furnishing milk to the city; that breeders should be taught the great danger of breeding tuberculous animals. Closing, he advised a more thorough education of the people to the dangers and the plans that give the greatest immunity from its dangers.

The subject was discussed at great length by many others, after which the Board of Trade adopted resolutions calling attention to the defective system existing for the production and sale of milk, and that the City Council should take the necessary steps looking to the establishment of a thorough and systematic inspection of milk dairies and dairy herds under the Health Department, and all vendors of milk should be licensed.

---

Philadelphia Milk Inspectors condemned some 527 quarts of milk during April as watered, colored, or skimmed.

